# Supercritical Fluid Extraction and Chromatography of Lipids in Bilberry

**DOI:** 10.1007/s11746-015-2680-x

**Published:** 2015-07-11

**Authors:** Firas Jumaah, Margareta Sandahl, Charlotta Turner

**Affiliations:** Department of Chemistry, Centre for Analysis and Synthesis, Lund University, P.O. Box 124, 22100 Lund, Sweden

**Keywords:** Bilberry, Supercritical fluid extraction, Supercritical fluid chromatography, GC–MS, Lipids, Fatty acids

## Abstract

A supercritical fluid extraction (SFE) method has been developed for the extraction of lipids in bilberry. Experimental design was used to optimize pressure, temperature and extraction time using CO_2_ as solvent. Best SFE condition for total lipids was 450 bar, 60 °C and 45 min. The SFE method was compared to conventional Bligh & Dyer (B&D) extraction. The amount of fatty acid methyl esters (FAME) was found to be 4.84 ± 0.06 mg and 4.564 ± 0.003 mg per g of the freeze-dried bilberry sample for the developed SFE and B&D methods, respectively, while the amount of total lipids was found to be 54.40 ± 6.06 mg and 65.70 ± 0.67 mg per g of sample for SFE and B&D, respectively. This discrepancy between FAME and total lipids could be explained by the presence of wax esters, sterol esters, carotenoids and phospholipids, as determined by supercritical fluid chromatography.

**Electronic supplementary material:**

The online version of this article (doi:10.1007/s11746-015-2680-x) contains supplementary material, which is available to authorized users.

## Introduction

Bilberry (*Vaccinium myrtillus* L.) is grown in central and northern Europe, northern Asia, and North America [1]. It is an important nutritional resource for both humans and animals. In Europe, bilberry is also known as European blueberry that make people confuse between them. Actually, bilberry and blueberry are not the same fruits. However, the easy way to distinguish between them is that bilberry is smaller and darker than blueberry; also, the bilberry pulp color is blue, whereas blueberries' pulp color is greenish. Other names of bilberry are whortleberry, blaeberry, hurtleberry, huckleberry, and winberry. Bilberry became widely known due to its potential health benefits. It contains polyphenols in high quantities such as anthocyanins [2] that may play an important role as antioxidants [3] with anti-inflammatory properties [4]. Recent scientific studies demonstrated that bilberry could be used to prevent colon cancer [5] and cardiovascular disease [6].

Extensive research has been carried out regarding the chemical composition of bilberry. Most attention has been focused on antioxidants in general [7], anthocyanins [2], and phenolic compounds [8] while the data about the lipid profile is limited [9]. Bunea *et al*. used a modified Folch method to extract total lipids from bilberry [9]. The average total lipids content was 650 mg per 100 g of fresh fruit. The main fatty acids (FA) extracted were polyunsaturated fatty acids (PUFA) such as linoleic (18:2) and linolenic (18:3) acids. PUFA have health benefits in the prevention of diseases such as cardiovascular disorders [10] and diabetes [11]. It has been shown that neat supercritical carbon dioxide (SC-CO_2_) is good for the extraction of neutral lipids, whereas a co-solvent such as ethanol is needed in order to extract polar lipids such as phospholipids [12].

In this study, SC-CO_2_ was used as an extraction solvent to extract total lipids from bilberry. SC-CO_2_ is useful as a “green” alternative solvent since it can dissolve similar compounds as hexane or heptane does, it is recyclable, inert and nonflammable, it has low toxicity, and is inexpensive.

To our knowledge, there is no study on the use of supercritical fluid extraction (SFE) to extract lipids from bilberry. The aims of this study were (1) to investigate an extraction method for lipids in bilberry using SC-CO_2_ as a green solvent; (2) to analyze the FA composition of bilberry using gas chromatography–mass spectrometry (GC–MS); (3) to analyze the lipid classes using supercritical fluid chromatography–mass spectrometry (SFC–MS) to provide more information about the lipid profile in bilberry; and (4) to compare the results with those obtained by the conventional solvent extraction method.

## Experimental Procedures

### Materials

High purity FAME were purchased from Sigma-Aldrich (St. Louis, MO, USA), ethanol 99.7 % from Solveco (Rosersberg, Sweden), *n*-heptane and chloroform from Merck (Darmstadt, Germany), boron trifluoride methanol complex from Schuchardt (85662, Hohenbrunn, Germany), methanol from B&J Brand (Honeywell, Seelze, Germany) and sodium chloride from Fisher Scientific UK (Bishop Meadow Road, Loughborough).

### Bilberry Sample Pre-treatment

Bilberries were purchased from a local market, thoroughly washed with tap water and rinsed with deionized water. The bilberries were blended well using a mortar and pestle, frozen at −18 °C for 24 h, and dried using a freeze-dryer (Hetosicc, Heto Birkerød Denmark) for 48 h. The dried bilberry was ground in a dry mill (Mill MM400, Retsch, Hann, Germany) to form a powder in order to increase the surface area of the sample, and then kept away from light in a freezer (at –18 °C) until analysis.

### SFE of Lipids from Bilberry

**Fig. 1:**
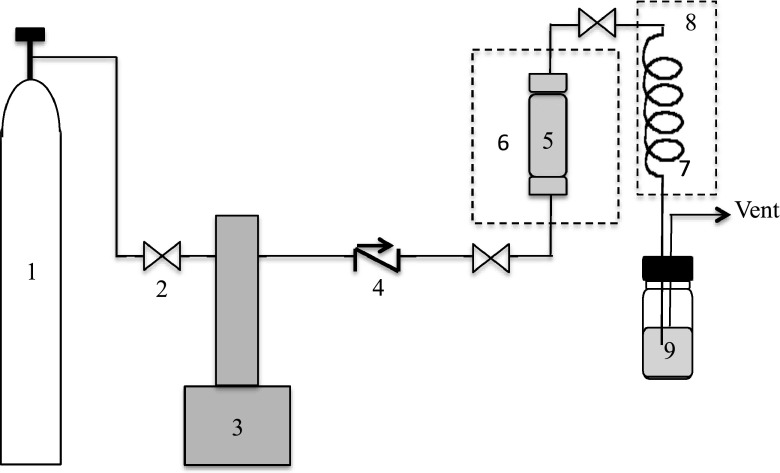
Scheme of the SFE equipment: (*1*) Liquid CO_2_ cylinder with a dip tube; (*2*) valve; (*3*) CO_2_ syringe pump (model ISCO 260 D, Teledyne ISCO, Thousand Oaks, CA) with a cooling jacket; (*4*) check valve; (*5*) extraction vessel (10 ml); (*6*) extraction oven (SFX 2-10); (*7*) restrictor; (*8*) heater; and (*9*) collection vial with solvent

SFE extraction experiments were carried out using SC-CO_2_ as a solvent. A scheme of the ISCO SFE apparatus is presented in Fig. 1.

One gram of freeze-dried bilberry was mixed with glass beads (3.0 mm diameter) and placed in the vessel, then 1 ml of ethanol as co-solvent was added directly to the vessel. Two extraction steps were conducted. First, static extraction was performed for 5 min and then, the dynamic extraction was started. Other conditions such as pressure, temperature, and dynamic extraction time were set according to the experimental design. The SC-CO_2_ flow rate was 1 ml/min. In the collection vial, 1 ml of ethanol was used as a trap solvent, which was removed at the end of each run under a gentle stream of nitrogen. The solid extracts were weighed and stored in a freezer at −18 °C.

### Design of Experiments

Three operating variables, pressure, temperature, and dynamic extraction time were studied as independent variables to determine the optimal conditions for the extraction of total lipids from bilberry using a central composite design (CCD). The dependent variables were the total lipids and FAME, in mg/g freeze-dried bilberry. The experimental ranges and levels are presented in Table 1. The number of experiments was 18, and four of them were replicated at the middle level of the operating independent variables.

**Table 1:** Experimental ranges and levels of operating variables

Operating variable	Coded levels		
	−1	0	1
Pressure (bar)	250	350	450
Temperature ( °C)	40	50	60
Dynamic extraction time (min)	20	40	60

### Conventional Extraction

Lipids were extracted from freeze-dried bilberry according to the method of Bligh and Dyer [13]. One gram of freeze-dried bilberry was vortex-mixed with 9.5 ml of a solvent mixture consisting of chloroform/methanol/water 1:2:0.8 (v/v/v) and subsequently diluted with chloroform and water to achieve two solvent layers. The final ratio of the solvent mixture (chloroform/methanol/water) was 2:2:1.8 (v/v/v). The mixture was centrifuged and the lower layer was transferred to a new test tube, dried under a stream of nitrogen and stored in a freezer at −18 °C until analysis.

### Hydrolysis and Derivatization of Neutral Lipids

Neutral lipids were hydrolyzed and converted to FAME according to Negh-Ngwainbi *et al*. [14]. This method consists of two steps. The first step was alkaline hydrolysis of the acylglycerols with 0.5 M sodium hydroxide in methanol, and the second step was an acidic *trans*-methylation reaction catalyzed by a BF_3_ reagent. A solution of saturated sodium chloride was added to facilitate phase separation. Centrifugation was applied to obtain a clear separation between the phases. The upper phase, containing the FAME, was transferred to a new vial, and methyl heptadecanoate was added as internal standard (IS). The sample was diluted to 10 ml with *n*-heptane and the concentration of IS after dilution was 0.05 mg/ml.

### GC–MS Analysis of FAME

Analysis of FAME was carried out with a Bruker GC–MS system (Bruker 450-GC, Netherlands and MS from Bruker Daltonics Inc., 3500 West Warren Avenue, Fremont, CA 94538, USA). The system was equipped with an autosampler (CP-8490, Malaysia), and a SCION TQ/SQ data system. FAME were separated on a capillary column (VF-23 ms, 30 m × 0.25 mm i.d., 0.25 µm), and the column temperature program was 65 °C held for 1 min, 5 °C/min to 120 °C, held for 4 min, followed by 5 °C/min to 230 °C, and held for 5 min. Helium was used as the carrier gas and the flow rate was 1.0 ml/min. Split injection was performed, the split ratio was 50, the injection volume was 1 µL, and the injector temperature was 250 °C. The MS detection conditions were as follows: full scan mode mass range 50–450 amu, EI^+^; electron energy, 70 eV; interface temperature 200 °C.

### SFC-MS Analysis of Lipid Classes

A Waters Acquity Ultra Performance Convergence Chromatography (UPC^2^) system was used for the analysis of lipid classes (Waters, Milford, MA, USA). CO_2_ was used as mobile phase A and methanol containing 2 g/L ammonium formate was used as mobile phase B. The analysis was performed using a Waters Acquity UPC^2^ HSS C18 SB column (100 mm × 3 mm, 1.8 µm) at 35 °C and the automated backpressure regulator (ABPR) was set to 200 bar. The flow rate was 1 ml/min and the injection volume was 1 µL. The gradient elution program employed is shown in Table 2. The column was re-equilibrated under initial conditions for 4 min before the next injection. The UPC^2^ system was coupled to a Waters XEVO-G2 Q-TOF (Quadruple-Time of Flight) mass spectrometer (Milford, MA, USA) for the analysis of lipid classes. The data acquisition was done in both positive and negative ion electrospray ionization (ESI) modes. The positive mode was run under the following conditions: data acquisition range was *m*/*z* 50–1200; capillary voltage 3.5 kV; cone voltage 35 V; source temperature 120 °C; desolvation temperature, 450 °C; cone gas flow rate 70 L/h; and desolvation gas flow rate 450 L/h. The conditions of negative mode was as following: data acquisition range was *m*/*z* 50–1200; capillary voltage 2.5 kV; cone voltage 20 V; source temperature 120 °C; desolvation temperature 650 °C; cone gas flow rate 70 L/h; and a desolvation gas flow rate of 450 L/h. The identification of some of the lipids was done by comparing the retention times and mass spectra with those obtained from standards. The remaining lipids were identified by using Waters MassLynex 4.1 software with the aid of ChemSpider. The identification was further confirmed with mass spectra of those found in the literature.

**Table 2:** Gradient elution program applied for SFC-MS analysis of lipid classes

Time (min)	Mobile phase A (%)	Mobile phase B (%)
Initial	98	2
5	95	5
15	50	50
21	50	50
22	98	2
26	98	2

## Results and Discussion

### Effect of SFE Pressure, Temperature and Dynamic Extraction Time on the Extracted Amount of Total Lipids and FAME

The influence of SFE variables including pressure, temperature and dynamic extraction time on the amount of total lipids and FAME was investigated using a CCD. The results in Table S1 (in the supplementary material) showed that the experimental values were in agreement with the predicted values for the total lipid and the FAME extracts. According to the results in Table 3, the extracted amount of total lipids and FAME (mg/g freeze-dried sample) varied from 13.5 to 54.4 and from 0.27 to 4.83, respectively, with the different sets of conditions. The extracted amount of total lipids was increased from around 30 to over 50 mg/g freeze dried bilberry with increasing the pressure from 250 to 450 bar and the temperature from 40 to 60 °C. This result shows that increasing the density of SC-CO_2_ (in this case from 0.79 to 0.97 g/mL) generally leads to higher solubility of lipids and subsequently improved extraction yields. This result is in agreement with other studies on SFE of lipids [15]. Our results also show that the temperature had a positive influence on the amount of total lipids extracted probably because of increasing the vapor pressure of lipids. This effect was clearer at higher pressure, which is also in agreement with literature [15]. CCD plots for total lipids and FAME are shown in Figure S2 and S3, respectively, in the supplementary material.

**Table 3:** Experimental design (CCD) with results of total lipids and FAME (mg/g freeze dried bilberry sample) for extracts in the separate SFE runs

Run#	*P* (bar)	*T* ( °C)	*t* (min)	CO_2_ density (g/ml)	Total lipids (mg/g)	FAME (mg/g)
1	250	40	20	0.88	13.5	1.75
2	250	40	60	0.88	30.6	2.91
3	250	60	20	0.79	22.7	1.47
4	250	60	60	0.79	38.3	4.83
5	450	40	20	0.97	26.4	0.46
6	450	40	60	0.97	36.8	2.44
7	450	60	60	0.91	54.4	1.91
8	450	60	20	0.91	45.0	0.27
9	250	50	40	0.83	39.9	2.02
10	450	50	40	0.94	34.8	2.73
11	350	40	40	0.93	42.9	0.96
12	350	60	40	0.86	47.5	2.44
13	350	50	20	0.90	15.6	1.98
14	350	50	60	0.90	34.0	1.92
15	350	50	40	0.90	29.0	1.32
16	350	50	40	0.90	21.3	0.47
17	350	50	40	0.90	35.5	2.04
18	350	50	40	0.90	26.5	2.53

The results in Table 3 show that there is a large difference between the extracted amounts of total lipids and FAME. This indicates that there is a large proportion of lipids not composed of mainly FA. This was further investigated, as described in later sections.

Analysis of variance (ANOVA) was applied to evaluate the adequacy of the fitted model (see Tables S4 and S5 in the supplementary material). The goodness of the fit of the model was checked by the determination coefficient (*R*^2^). The *R*^2^ values for total lipids and FAME were 0.80 and 0.60, respectively, indicating that the model was able to explain 80 % of the results for total lipids and 60 % of the results for FAME with no significant lack of fit at *p* > 0.05 in both cases. *p* values were used to determine the significance of each coefficient. According to the results in Table S6 in the supplementary material, the main effect of temperature and dynamic extraction time (*T* and *t*) had significant (*p* < 0.05) effects on the extracted amount of total lipids. T^2^ was significant at the level of *p* < 0.05, whereas the interaction effects (PT, Pt, and Tt) were insignificant (*p* > 0.05). The linear, interactions, and quadratic effects on extracted amount of FAME were insignificant (*p* > 0.05), except the linear effect of time (t) that had significant (*p* < 0.05) effect on the amount of FAME, see Table S7 in the supplementary material. Main effects and interaction effects for total lipid recovery are shown in Figure S8 (supplementary material).

Within the range of investigated extraction conditions, the following SFE conditions were found to be best: 450 bar, 60 °C and 45 min dynamic extraction time for total lipids and 250 bar, 60 °C and 60 min dynamic extraction time for FAME, respectively. These are not optimum conditions, i.e., even higher pressure would have been beneficial for total lipids extraction, however, 450 bar was the maximum operating pressure for the SFE system.

### Comparison of SFE with a Conventional Extraction Method

The developed SFE method was compared with B&D as a conventional extraction method. Four replicate extraction experiments of lipids from freeze-dried bilberry were performed using SFE under optimized conditions for total lipids (450 bar, 60 °C and 45 min dynamic extraction), whereas three replicates were carried out using B&D. Total lipids were found to be 65.70 ± 0.67 mg/g freeze-dried bilberry using the B&D method and 54.40 ± 6.06 mg/g freeze-dried bilberry for the SFE method (mean ± SD). The difference between the SFE and the B&D in terms of gravimetric results could be due to the fact that the B&D method is more efficient for the extraction of polar lipids such as phospholipids in comparison with the SFE method. In the SFE method, 1 ml of ethanol was added directly to the extraction vessel as a co-solvent in order to improve the initial stage of the extraction and to enhance the solubility of polar lipids such as phospholipids in SC-CO_2_. The limitation of only adding the co-solvent to the extraction vessel rather than continuously to the SC-CO_2_ is that the effect of ethanol ended as soon as it was removed from the extraction vessel by SC-CO_2_ [16].

Although these gravimetric results show that the B&D method is better than the SFE method in terms of extraction efficiency of total lipids and precision, the SFE method was considered to be “greener” in comparison to the B&D method since the latter is based on using chloroform as an extraction solvent. Furthermore, the question is which compounds are extracted using the two separate methods. Gravimetric analysis does not give information about the chemical composition. Results described above also show that there is a large discrepancy between the extracted amount of FAME (based on GC–MS analysis after derivatisation of the lipid extract) and extracted amount of total lipids (gravimetric data) using the SFE method (Table 3). For this reason, lipid extracts obtained with the optimized SFE method and the B&D method were derivatized to FAME and analyzed by GC–MS.

### Hydrolysis and Derivatization of Lipids

A derivatization step is needed to convert FA-containing lipids in the bilberry extracts to FAME that are volatile enough for GC–MS analysis. At the beginning, Metcalfe’s method [17] was used for derivatization of bilberry extracts. In this method, the sample and all reagents were put into a test tube with a cap, the cap was tied well and the test tube was heated in an oil bath. The main problem of this method was that a high pressure was generated inside the tube, which caused evaporation of a small amount of sample and reagents. A test tube even burst during the run of experiments. The second derivatization method was that of Palmquist *et al*. [18]. The conditions of this method are similar to the method of Metcalfe, resulting in similar problems with build-up of pressure. Finally, a method of Ngeh-Ngwainbi *et al*. [14] was tested. In this method, the sample was transferred to a round bottle flask by a small amount of *n*-heptane, the flask was attached to a water-cooled condenser, heated on a hot plate heater, and all the reagents were added through the top of the condenser to avoid evaporation and losing of the sample and reagents. No problems occurred during the experiment. The efficiency of this method was studied by hydrolysis and derivatization of a standard, triolein, and the amount of produced methyl oleate was determined. The results showed that this method of Ngeh-Ngwainbi *et al*. was efficient enough, giving an average recovery of 95 % and an RSD of 8.41 % (*n* = 4).

### GC–MS Analysis of FAME

**Fig. 2:**
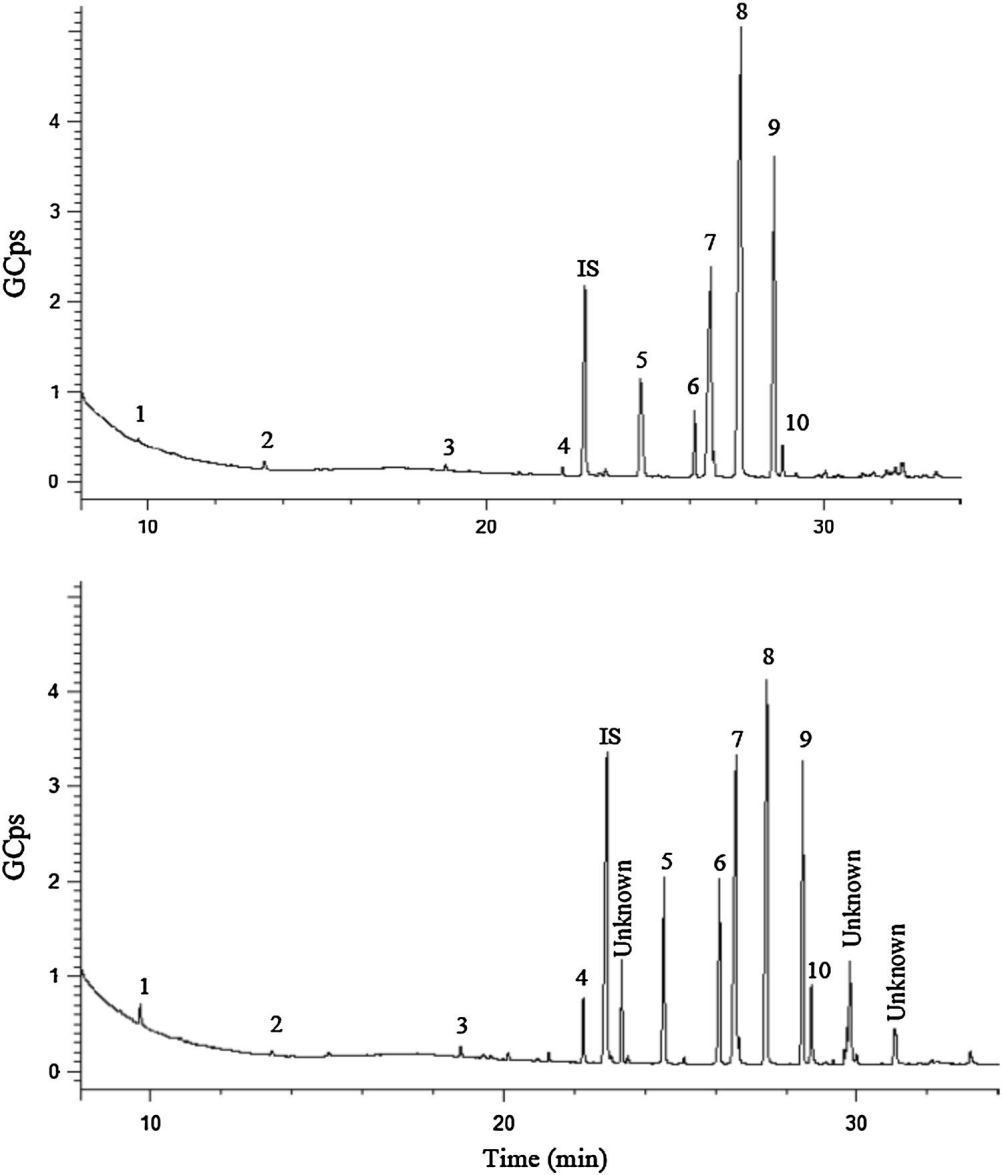
GC–MS chromatograms for analysis of extracted FAME from bilberry using the B&D method (*top chromatogram*) and the optimized SFE method (*bottom chromatogram*). The internal standard (IS) was methyl heptadecanoate. Peak identification is found in Table 4

FAME were analyzed and separated using a Bruker GC–MS system in the full-scan acquisition mode. A representative chromatogram is shown in Fig. 2. FAME were identified by comparing their retention times and mass spectra with those obtained from injected standards. In Table 4, the profile and percentage contents of FAME extracted from bilberry by the SFE and the B&D methods are compared. It is obvious that the individual FAME extracted by both the SFE and the B&D methods were quite similar, although a slightly higher recovery of lipids containing saturated fatty acid was observed using SFE, and the opposite could be observed for the highly unsaturated linolenic acid. The main FAME extracted from bilberry using both extraction methods were methyl palmitate, methyl oleate, and methyl linolenate. In addition, the total FAME extracted amount from freeze-dried bilberry were almost the same using SFE (4836 ± 56 µg/g) and B&D (4564 ± 3 µg/g), which means that the results of the SFE method agreed well with the B&D method concerning the extraction of FA-derived lipids. However, as mentioned previously, there is a large difference between the extracted amount of total lipids and FAME using both the SFE and B&D methods. In addition, a higher amount of total lipids was recovered by the B&D method. Therefore, SFC-QTOF-MS was carried out for analysis of lipid classes in bilberry extracts in order to understand the reasons for such differences.

**Table 4:** FAME identified in bilberry extracts obtained by the optimized SFE method and the B&D method. Analyses were done by GC-MS

Peak	Fatty acid methyl ester	FAME (µg/g freeze-dried bilberry)^a^		FAME (w/w %)	
		SFE	B&D	SFE	B&D
1	Methyl decanoate (C10:0)	118 ± 3.3	53.8 ± 0.1	2.44	1.18
2	Methyl dodecanoate (C12:0)	15.0 ± 0.4	2.9 ± 0.0	0.31	0.06
3	Methyl myristate (C14:1)	36.3 ± 0.1	26.2 ± 0.3	0.75	0.57
4	Methyl palmitate (C16:0)	1229.4 ± 48.2	688.0 ± 0.9	25.42	15.07
5	Methyl stearate (C18:0)	495.9 ± 4.5	175.4 ± 0.7	10.25	3.84
6	Methyl oleate (C18:1)	1242.2 ± 13.6	1200.5 ± 2.1	25.69	26.31
7	Methyl linoleate (C18:2)	66.3 ± 0.5	70.5 ± 0.1	1.37	1.54
8	Methyl linolenate (C18:3)	1115.5 ± 6.7	2247.9 ± 2.6	23.07	49.26
9	Methyl eicosanoate (C20:0)	215.5 ± 4.6	89.0 ± 0.2	4.46	1.95
10	Cis-11,14,17-Eicosatrienoic acid methyl ester (C20:3)	301.4 ± 0.6	9.9 ± 0.0	6.23	0.22
	Total FAME	4836 ± 56	4564 ± 3		

^a^Mean ± SD

### SFC-MS Analysis of Lipid Classes

**Fig. 3:**
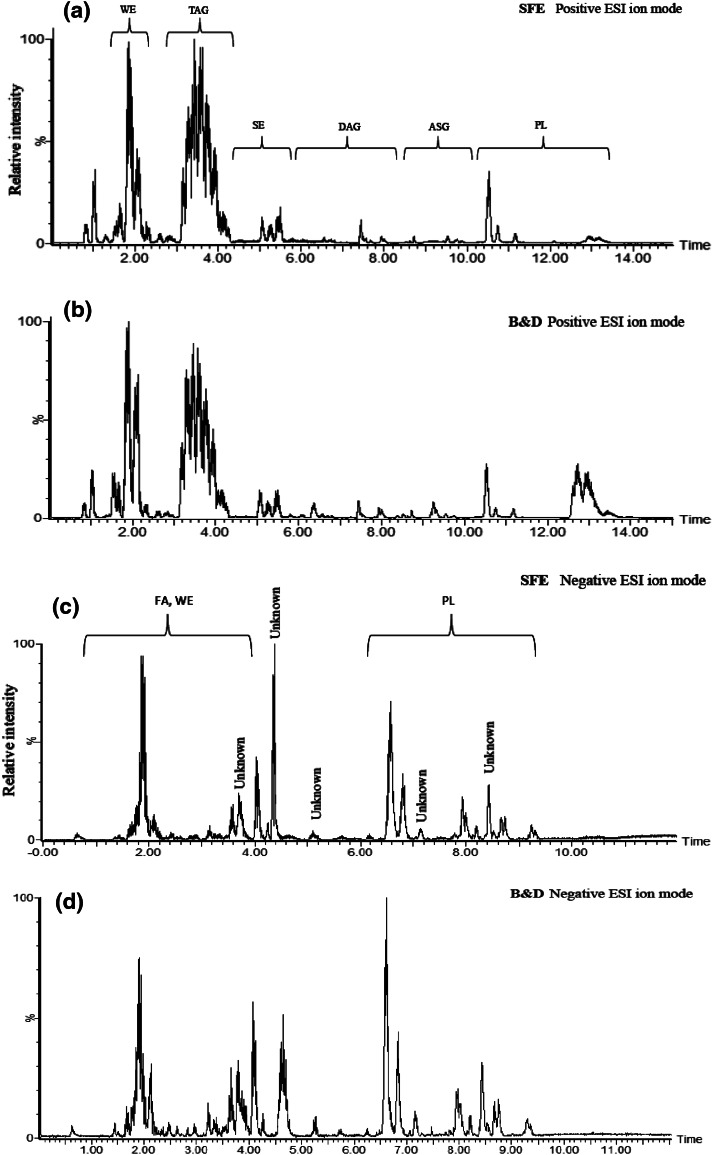
SFC-QTOF-MS chromatograms of total lipids extracts using ESI in positive mode (SFE: Figure **a**, B&D: Figure **b**) and in negative mode (SFE: Figure **c**, B&D: Figure **d**)

Information on lipid classes in bilberry is limited, thus one aim of this study was to analyze the lipid classes in order to provide more information about lipid composition in bilberry. Hence, lipids were extracted from bilberry using both the SFE and the B&D extraction methods and then analyzed by SFC-QTOF-MS. ESI in both positive and negative modes were used in order to enable efficient ionization of both polar and non-polar lipids. SFC chromatograms for total lipids extracts obtained by SFE are shown in Fig. 3. Interestingly, it appears that B&D lipid extracts contain relatively larger amounts of phospholipids (PL) than extracts produced by SFE. This could explain the higher amount total lipids extracted by B&D as discussed above.

The major lipids identified in bilberry extracts were triacylglycerols (TAG), diacylglycerols (DAG), wax esters (WE), sterol esters (SE), acylated sterol glucosides (ASG) and PL as presented in Tables S9 and S10. Although more than 200 peaks were detected in the extracts, only the most abundant lipid compounds were identified. According to the results in Table S9 and S10, the most abundant ions are derived from the TAG, SE and PL lipid classes. The *m*/*z* values for the [M + NH_4_]^+^ ion of the TAG were 890.7304, 892.7424, 894.7424, 896.7730, 898.7856, 900.8005 and 902.8176. Figure S11 shows the ion of [M + NH_4_]^+^ at *m*/*z* 902.8176, which refers to TAG consisting of stearic acid (S), oleic acid (O) and linoleic acid (L). The [DAG]^+^ fragments were 601.3646, 603.5411 and 605.5501, which corresponds to the diacylglycerol fragments [O–L]^+^, [S–L]^+^ and [S–O]^+^, respectively. SE were recognized to form [M + NH_4_]^+^ ion and fragmentation ions such as 369.3346, 383.3658 and 397.3851, which corresponded to [cholesterol-H_2_O]^+^, [campesterol-H_2_O]^+^ and [sitosterol-H_2_O]^+^, respectively (see Figure S12 in the supplementary material). PL were detected in both positive and negative ionization modes. Phosphatidylcholine (PC), phosphatidylethanolamine (PE), phosphatidylinositol (PI), phosphatidylserine (PS), lysophosphatidylglycerol (LPG), and lysophosphatidylinositol (LPI), which belong to PL classes, were identified and are presented in Tables S9 and S10. The most abundant PL were PC and PE. Figure S13 shows the mass spectra of PC (34:2). The ion with highest abundance is the protonated molecule [M + H]^+^ with an *m*/*z* 758. 5724 and the *m*/*z* of the polar head group is 184.0754. Further, carotenoids, WE, DAG, and free fatty acids were also identified in the bilberry extracts. For instance, Figure S14 shows MS data of lutein.

## Conclusions

An SFE method based on the use of neat SC-CO_2_ has been optimized by studying the effects of pressure, temperature, and dynamic extraction time on total lipids and FAME in bilberry using CCD experimental design. The highest recovery of total lipids was accomplished at 450 bar, 60 °C and 45 min dynamic extraction time. The SFE method was compared to B&D, demonstrating the feasibility of the former method in terms of speed and greenness. However, the obtained extracts from SFE and B&D differ in chemical composition with respect to for instance PL. Furthermore, alkaline hydrolysis with BF_3_-catalyzed methylation followed by GC–MS analysis of the produced FAME severely underestimates the lipid content in bilberry. SFC-MS analysis of lipid classes shows that bilberry contains significant amounts of carotenoids as well as WE, SE and PL that are likely not as easily hydrolyzed and methylated as neutral acyl glycerols.

## Electronic supplementary material

Supplementary material 1 (PDF 3312 kb)
