# Supplementary material for: Supercritical Fluid Extraction and Chromatography of Lipids in Bilberry
Source: J Am Oil Chem Soc. 2015 Jul 11;92(8):1103–11. doi: 10.1007/s11746-015-2680-x (PMC4522036; doi:10.1007/s11746-015-2680-x)
Supplement: Supplementary file 1 — Supplementary material 1 (PDF 3312 kb) [file 11746_2015_2680_MOESM1_ESM.pdf]

Supporting Information

Supercritical Fluid Extraction and Chromatography of Lipids in Bilberry

Firas Jumaah, Margareta Sandahl and Charlotta Turner

Content:

Table S1. Experimental design of CCD with experimental and predicted values of total lipid and FAMEs extracts of each run.

Figure S2. Central composite design (CCD) plot for the SFE optimization showing the effects of pressure, temperature and time on extracted amount of total lipids (mg per g of freeze-dried bilberry).

Figure S3. Central composite design (CCD) plot for the SFE optimization showing the effects of pressure, temperature and time on extracted amount of FAMEs (mg per g of freeze-dried bilberry).

Table S4. ANOVA for the fitted quadratic polynomial model for SFE of total lipids.

Table S5. ANOVA for the fitted quadratic polynomial model for SFE of FAMEs.

Table S6. Estimated regression model of relationship between the total lipid and independent variables (P, T, and t).

Table S7. Estimated regression model of relationship between the FAMEs and independent variables (P, T, and t).

Figure S8. Main effects and interaction effects for pressure (x1), temperature (x2) and time (x3) on the extracted amount of total lipids (mg per g of freeze-dried bilberry).

Table S9. Lipids identified in positive mode for the SFE extracts.

Table S10. Lipids identified in negative mode for the SFE extracts.

Figure S11. MS spectra of a triacylglycerol (S-L-O) with fragmentation ions of diacylglycerols (O-L, S-L, S-O) in bilberry extracts. Abbreviations: S-stearic acid; O - oleic acid; L - linoleic acid.

Figure S12. MS spectra of sitosterol esters: 18:0-sitosterol (top spectra), (B) 18:1-sitosterol (mid spectra), and 18:2-sitosterol (bottom spectra) in bilberry extract.

Figure S13. MS spectra of phosphatidylcholine (16:0/18:2) in bilberry extract.

Figure S14. MS spectra of lutein in bilberry extract.

Table S1. Experimental design of CCD with experimental and predicted values of total lipid and FAMEs extracts of each run.

Run	P (bar)	T (°C)	t (min)	mg/g freeze-dried sample			
				Total lipids		FAMEs	
				Experimental	Predicted	Experimental	Predicted
1	250	40	20	13.5	16.7	1.75	1.74
2	250	40	60	30.6	34.7	2.91	2.32
3	250	60	20	22.7	24.0	1.47	1.35
4	250	60	60	38.3	40.8	4.83	4.84
5	450	40	20	26.4	25.6	0.46	0.66
6	450	40	60	36.8	37.2	2.44	2.51
7	450	60	60	54.4	52.9	1.91	2.01
8	450	60	20	45.0	42.6	0.27	0.94
9	250	50	40	39.9	28.7	2.02	2.73
10	450	50	40	34.8	39.2	2.73	1.70
11	350	40	40	42.9	36.1	0.96	1.30
12	350	60	40	47.5	47.6	2.44	1.78
13	350	50	20	15.6	14.3	1.98	1.38
14	350	50	60	34.0	28.5	1.92	2.20
15	350	50	40	29.0	31.5	1.32	1.75
16	350	50	40	21.3	31.5	0.47	1.75
17	350	50	40	35.5	31.5	2.04	1.75
18	350	50	40	26.5	31.5	2.53	1.75

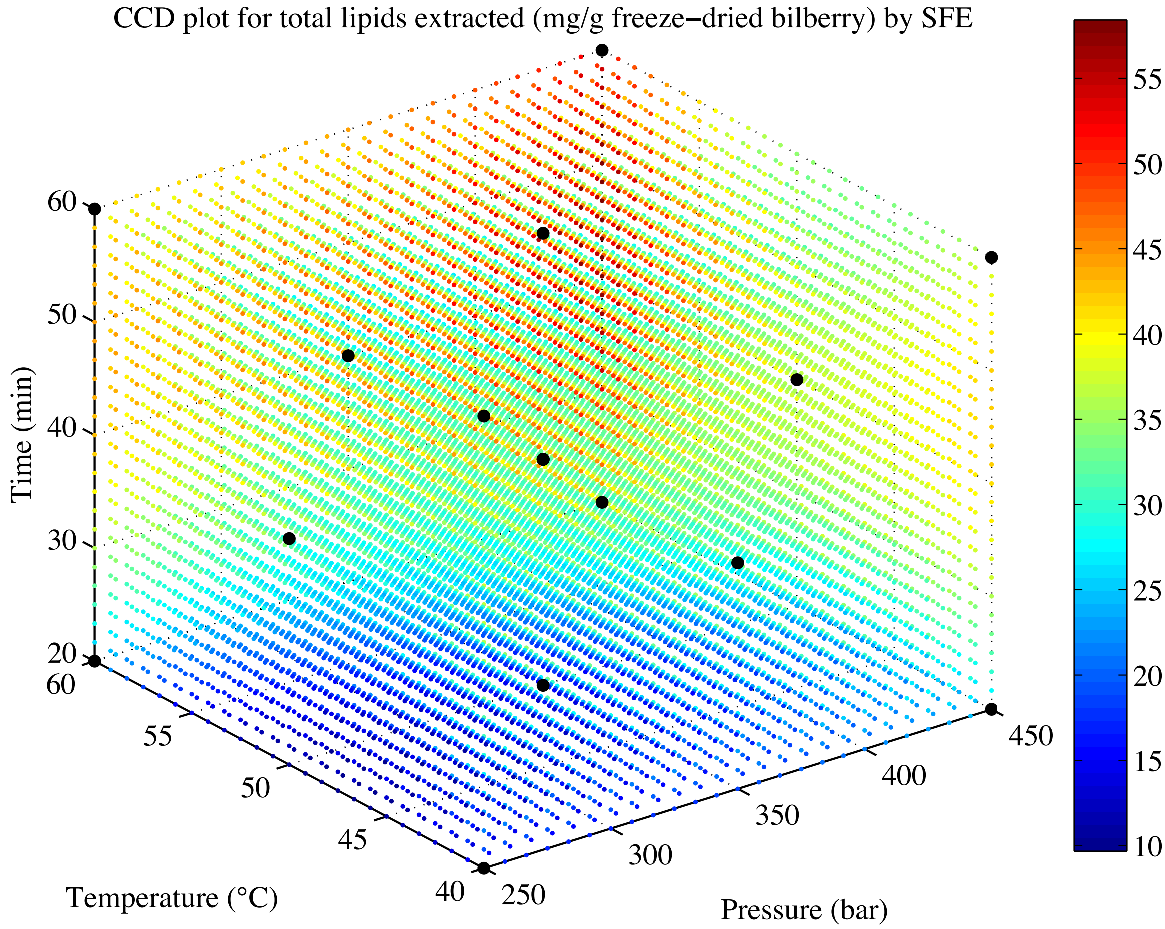

Figure S2. Central composite design (CCD) plot for the SFE optimization showing the effects of pressure, temperature and time on extracted amount of total lipids (mg per g of freeze-dried bilberry).

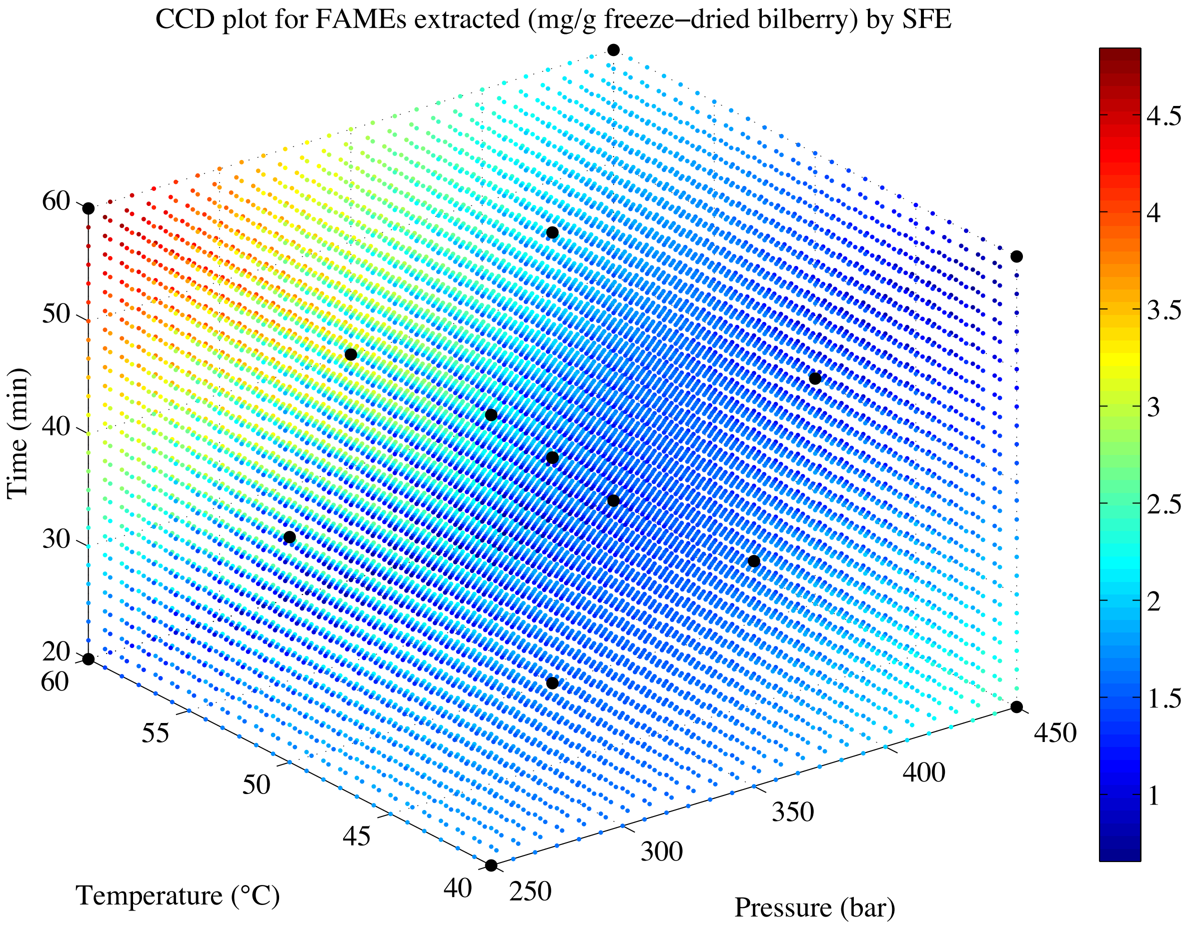

Figure S3. Central composite design (CCD) plot for the SFE optimization showing the effects of pressure, temperature and time on extracted amount of FAMEs (mg per g of freeze-dried bilberry).

Table S4. ANOVA for the fitted quadratic polynomial model for SFE of total lipids.

	Sum of squares	Degree of freedom	Mean of square	F-value	p-value
Total	2044.4	17	120.26		
Model	1627.2	9	180.8	3.4669	0.047054
Linear	1110.2	3	370.06	7.0962	0.012105
Nonlinear	517	6	86.167	1.6523	0.24964
Residual	417.2	8	52.15		
Lack of fit	312.83	5	62.566	1.7984	0.3336
Pure error	104.37	3	34.789		
R^2	0.80				
Adjusted R^2	0.57				

Table S5. ANOVA for the fitted quadratic polynomial model for SFE of FAMEs.

	Sum of squares	Degree of freedom	Mean of square	F-value	p-value
Total	19.514	17	1.148		
Model	11.744	9	1.305	1.343	0.344
Linear	9.778	3	3.259	3.356	0.076
Nonlinear	1.966	6	0.328	0.337	0.899
Residual	7.770	8	0.971		
Lack of fit	5.357	5	1.071	1.332	0.433
Pure error	2.413	3	0.804		
R^2	0.60				
Adjusted R^2	0.15				

Table S6. Estimated regression model of relationship between the total lipid and independent variables (P, T, and t). Second order polynomial equation: $y = 0.00175 - 0.0108P + 0.19175T - 0.0630t - 0.0003PT - 0.0003\ Pt + 0.00364\ Tt + 0.00005\ P^2 - 0.0021\ T^2 + 0.0001\ t^2$, where y is the total amount of total lipids (mg/g) extracted from freeze-dried bilberry. P, T, and t are pressure, temperature and dynamic extraction time, respectively.

	Sum of squares	Degree of freedom	Mean of square	F-value	p-value
P	274.58	1	274.58	5.2652	0.050898
T	332.93	1	332.93	6.3841	0.035438
t	502.68	1	502.68	9.6392	0.014562
PT	46.561	1	46.561	0.89284	0.37237
Pt	20.801	1	20.801	0.39888	0.54529
Tt	0.78125	1	0.78125	0.014981	0.9056
P^2	17.178	1	17.178	0.3294	0.58178
T^2	291.27	1	291.27	5.5853	0.045721
t^2	272.71	1	272.71	5.2294	0.051522
Error	417.2	8	52.15		

Table S7. Estimated regression model of relationship between the FAMEs and independent variables (P, T, and t).

	Sum of squares	Degree of freedom	Mean of square	F-value	p-value
P	2.673	1	2.673	2.752	0.136
T	0.576	1	0.576	0.593	0.463
t	6.527	1	6.527	6.722	0.032
PT	0.696	1	0.696	0.717	0.422
Pt	0.101	1	0.101	0.104	0.755
Tt	0.432	1	0.432	0.445	0.523
P^2	0.586	1	0.586	0.603	0.460
T^2	0.119	1	0.119	0.123	0.735
t^2	0.004	1	0.004	0.004	0.948
Error	7.770	8	0.971		

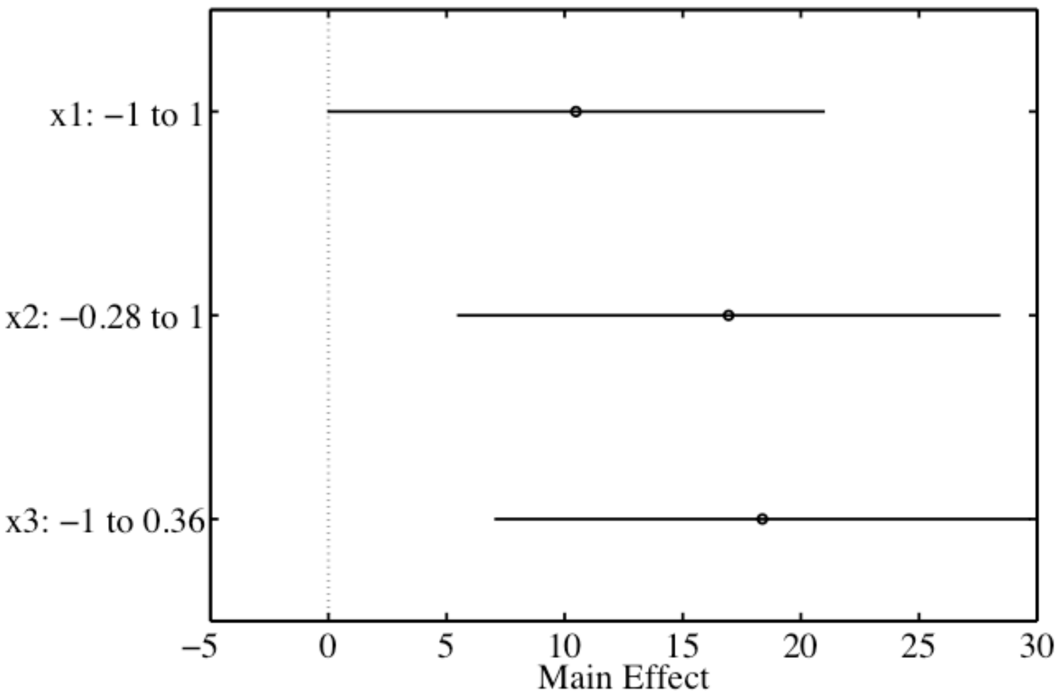

Figure S8 A. The main effects of pressure (x1), temperature (x2) and time (x3) on the extracted amount of total lipids (mg per g of freeze-dried bilberry).

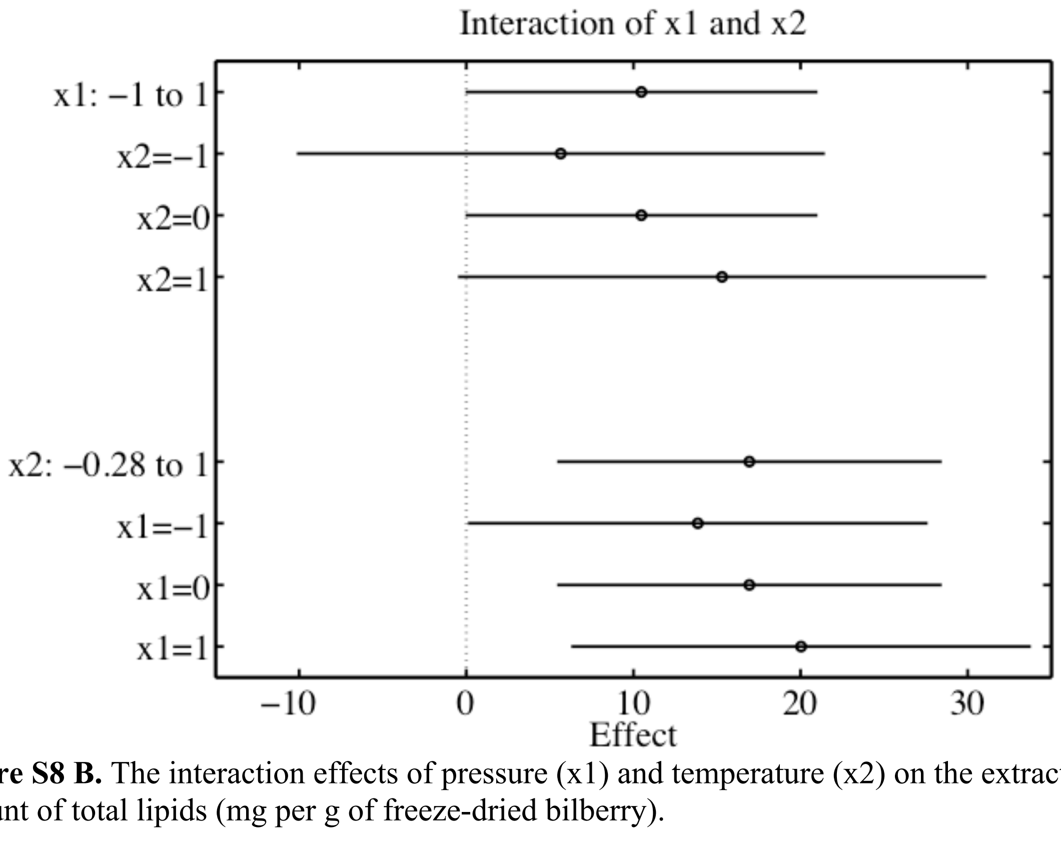

Figure S8 B. The interaction effects of pressure (x1) and temperature (x2) on the extracted amount of total lipids (mg per g of freeze-dried bilberry).

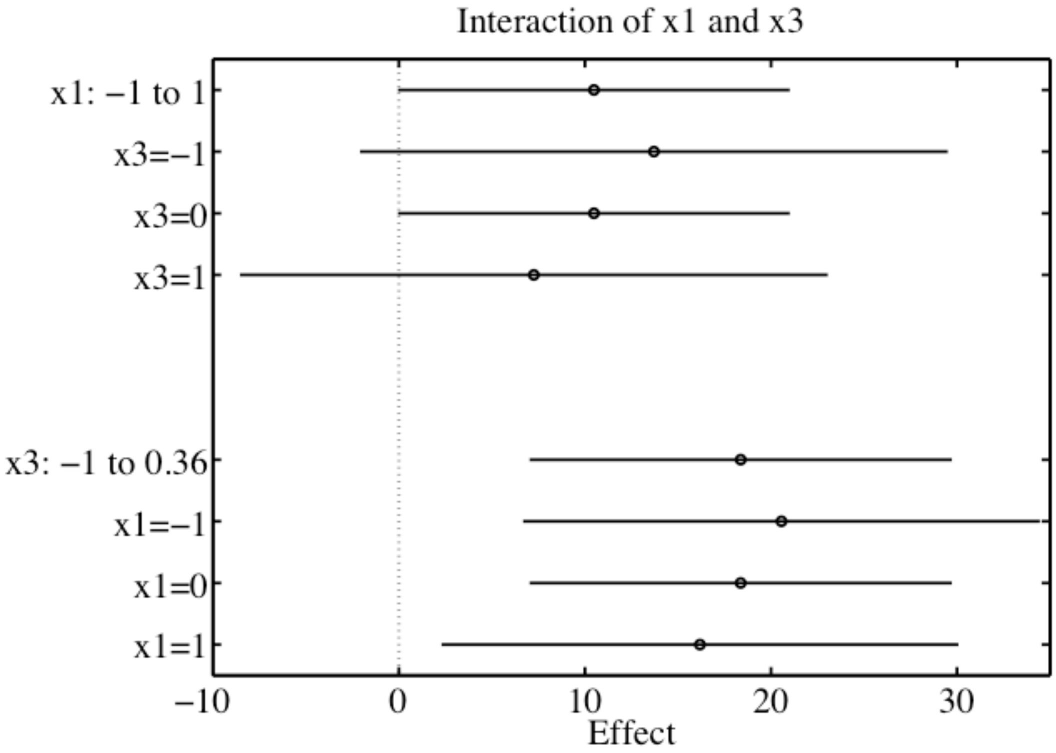

Figure S8 C. The interaction effects of pressure (x1) and time (x3) on the extracted amount of total lipids (mg per g of freeze-dried bilberry).

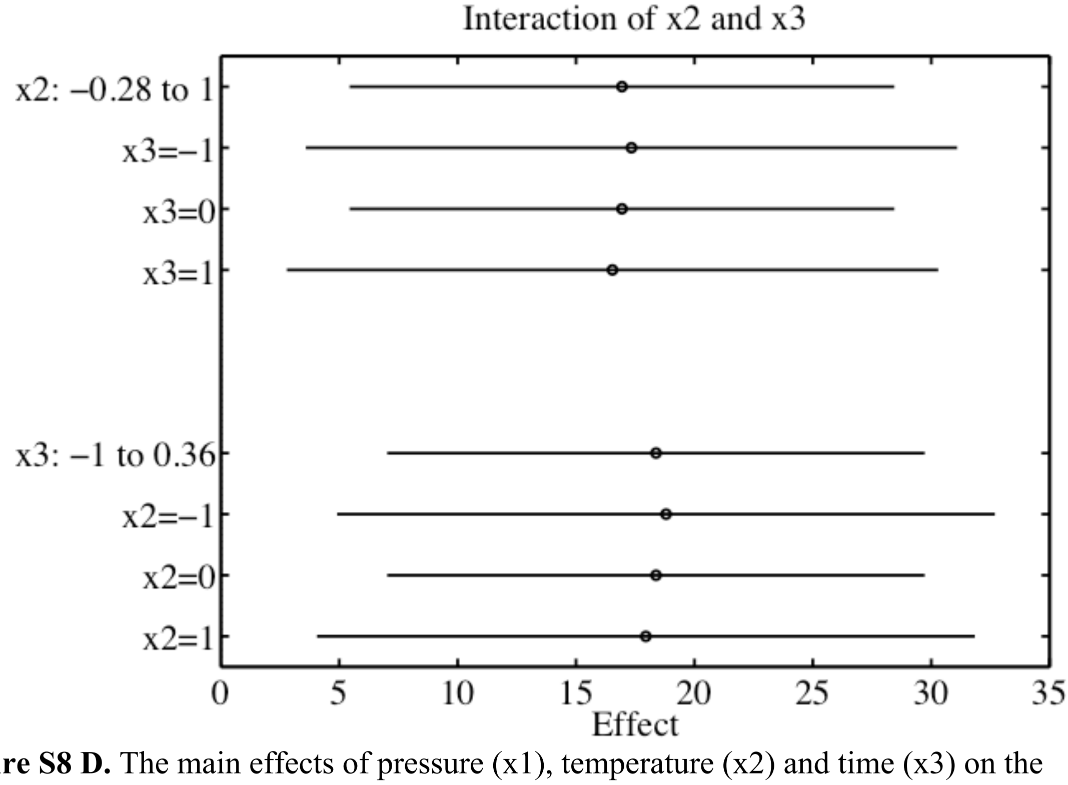

Figure S8 D. The main effects of pressure (x1), temperature (x2) and time (x3) on the extracted amount of total lipids (mg per g of freeze-dried bilberry).

Table S9. Lipids identified in positive mode for the SFE extracts.

RT (min)	Observed main m/z	Adduct type	Formula	Tentative compound class
1.027	391.2853	$[M+H]^+$	C24H38O4	Dioctyl phathate
1.643	437.4343	$[M+H]^+$	C29H56O2	WE 29:1
1.842	465.4654	$[M+H]^+$	C31H60O2	WE 31:1
2.276	449.4701	$[M+H]^+$	C30H56O2	WE 30:2
3.168	890.7304	$[M+NH_4]^+$	C56H103O6	TAG 53:2
3.284	892.7424	$[M+NH_4]^+$	C56H105O6	TAG 53:1
3.438	894.7565	$[M+NH_4]^+$	C57H99O6	TAG 54:7
3.575	896.773	$[M+NH_4]^+$	C57H101O6	TAG 54:6
3.74	898.7856	$[M+NH_4]^+$	C57H103O6	TAG 54:5
3.924	900.8005	$[M+NH_4]^+$	C57H105O6	TAG 54:4
4.141	902.8176	$[M+NH_4]^+$	C56H107O6	TAG 54:3
4.295	666.601	$[M+NH_4]^+$	C45H76O2	18:2-Cholesterol
5.033	678.6305	$[M+NH_4]^+$	C46H76O2	18:3-campesterol
5.196	680.6325	$[M+NH_4]^+$	C46H78O2	18:2-campesterol
5.276	692.6365	$[M+NH_4]^+$	C47H78O2	18:3-sitosterol
5.497	694.6525	$[M+NH_4]^+$	C47H80O2	18:2-sitosterol
5.812	696.6629	$[M+NH_4]^+$	C47H82O2	18:1-sitosterol
7.438	613.4826	$[M+Na]^+$	C37H66O5	DAG 34:3
7.49	615.4979	$[M+Na]^+$	C37H68O5	DAG 34:2
7.562	617.5166	$[M+Na]^+$	C37H70O5	DAG 34:1
7.948	736.5349	$[M+H-2H_2O]^+$	C42H78NO9P	PS 36:2
8.001	554.516	$[M+NH_4]^+$	C40H56	*β*-carotene
8.38	654.6005	$[M+NH_4]^+$	C40H76O5	DAG 37:1
8.537	682.6321	$[M+NH_4]^+$	C42H80O5	DAG 39:1
8.613	696.652	$[M+NH_4]^+$	C43H82O5	DAG 40:1
8.657	710.6835	$[M+NH_4]^+$	C48H84O2	20:1-campesterol
8.946	568.4277	$[M]^+$	C40H52O2	Lutein
9.372	840.6769	$[M+NH_4]^+$	C52H86O7	18:3-Glc-campesterol
9.422	854.6882	$[M+NH_4]^+$	C53H88O7	18:3-Glc-sitosterol
9.452	842.6851	$[M+NH_4]^+$	C52H88O7	18:2-Glc-campesterol
9.528	856.701	$[M+NH_4]^+$	C53H90O7	18:2-Glc-sitosterol
9.72	858.7102	$[M+NH_4]^+$	C53H92O7	18:1-Glc-sitosterol
9.741	931.594	$[M+H-2H_2O]^+$	C53H91O13P	PI 44:6
10.038	860	$[M+NH_4]^+$	C53H94O7	18:0- Glc-sitosterol
10.327	888.7635	$[M+NH_4]^+$	C55H98O7	20:0-Glc-sitosterol
10.667	916.7946	$[M+NH_4]^+$	C57H102O7	22:0-Glc-sitosterol
10.73	885.5551	$[M+H]^+$	C47H81O13P	PI 38:5
11.167	871.5761	$[M+H]^+$	C47H83O12P	PI 38:4
12.827	780.5572	$[M+H]^+$	C44H78NO8P	PC 36:5
12.926	782.5724	$[M+H]^+$	C44H80NO8P	PC 36:4
13.134	758.5724	$[M+H]^+$	C42H80NO8P	PC 34:2
13.58	786.6012	$[M+H]^+$	C44H84NO8P	PC 36:2
13.75	784.5871	$[M+H]^+$	C44H82NO8P	PC 36:3

Table S10. Lipids identified in negative mode for the SFE extracts.

RT (min)	Observed main m/z	Adduct type	Formula	Tentative compound class
1.648	435.4289	$[M-H]^-$	C29H56O2	WE 29:1
1.724	277.2245	$[M-H]^-$	C18H30O2	FA 18:3
1.778	279.239	$[M-H]^-$	C18H32O2	FA 18:2
1.84	463.4628	$[M-H]^-$	C31H60O2	WE 31:1
2.082	283.2691	$[M-H]^-$	C18H34O2	FA 18:0
2.399	311.3035	$[M-H]^-$	C20H40O2	FA 20:0
3.152	367.3677	$[M-H]^-$	C24H47O2	FA 24:0
3.598	395.3966	$[M-H]^-$	C26H51O2	FA 26:0
3.704	263.1345	-	-	Unknown
4.031	423.43	$[M-H]^-$	C28H55O2	FA 28:0
4.234	437.4464	$[M-H]^-$	C29H57O2	FA 29:0
4.366	451.4644	-	-	Unknown
5.103	479.4944	-	-	Unknown
6.575	455.3676	$[M-H]^-$	C20H41O9P	LPG 14:0
7.787	471.3622	-	-	Unknown
7.83	730.5461	$[M-H]^-$	C40H78NO8P	PE 35:1
7.931	758.5636	$[M-H]^-$	C40H78NO10P	PS 34:2
8.275	832.6418	$[M-H]^-$	C45H88NO10P	PS 39:0
8.397	531.4583	-	-	Unknown
8.73	621.4544	-	-	Unknown
9.182	736.5099	$[M-H]^-$	C41H72NO8P	PE 36:5
9.227	738.53	$[M-H]^-$	C39H72NO8P	PE 34:3
9.297	714.524	$[M-H]^-$	C39H74NO8P	PE 34:2

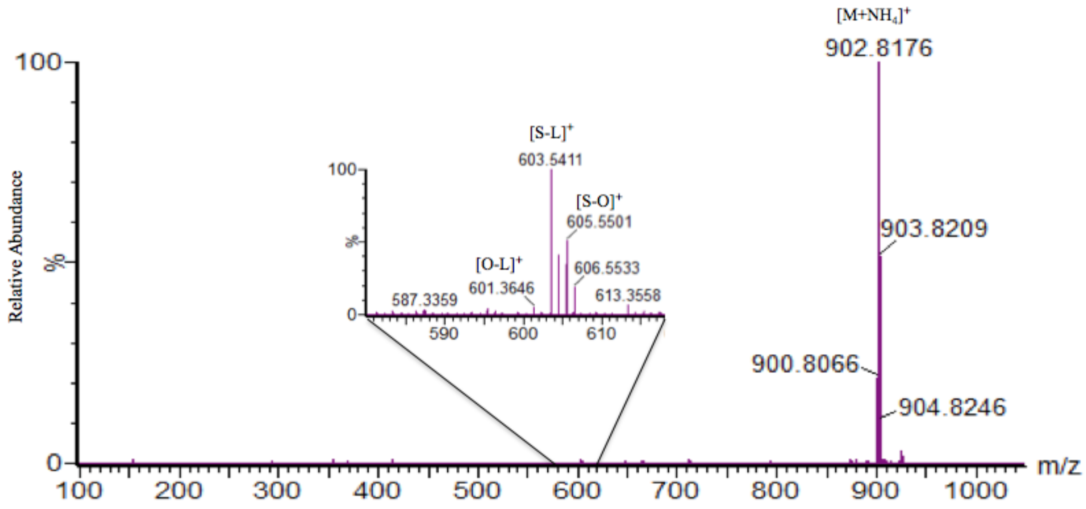

Figure S11. MS spectra of a triacylglycerol (S-L-O) with fragmentation ions of diacylglycerols (O-L, S-L, S-O) in bilberry extracts. Abbreviations: S-stearic acid; O - oleic acid; L - linoleic acid.

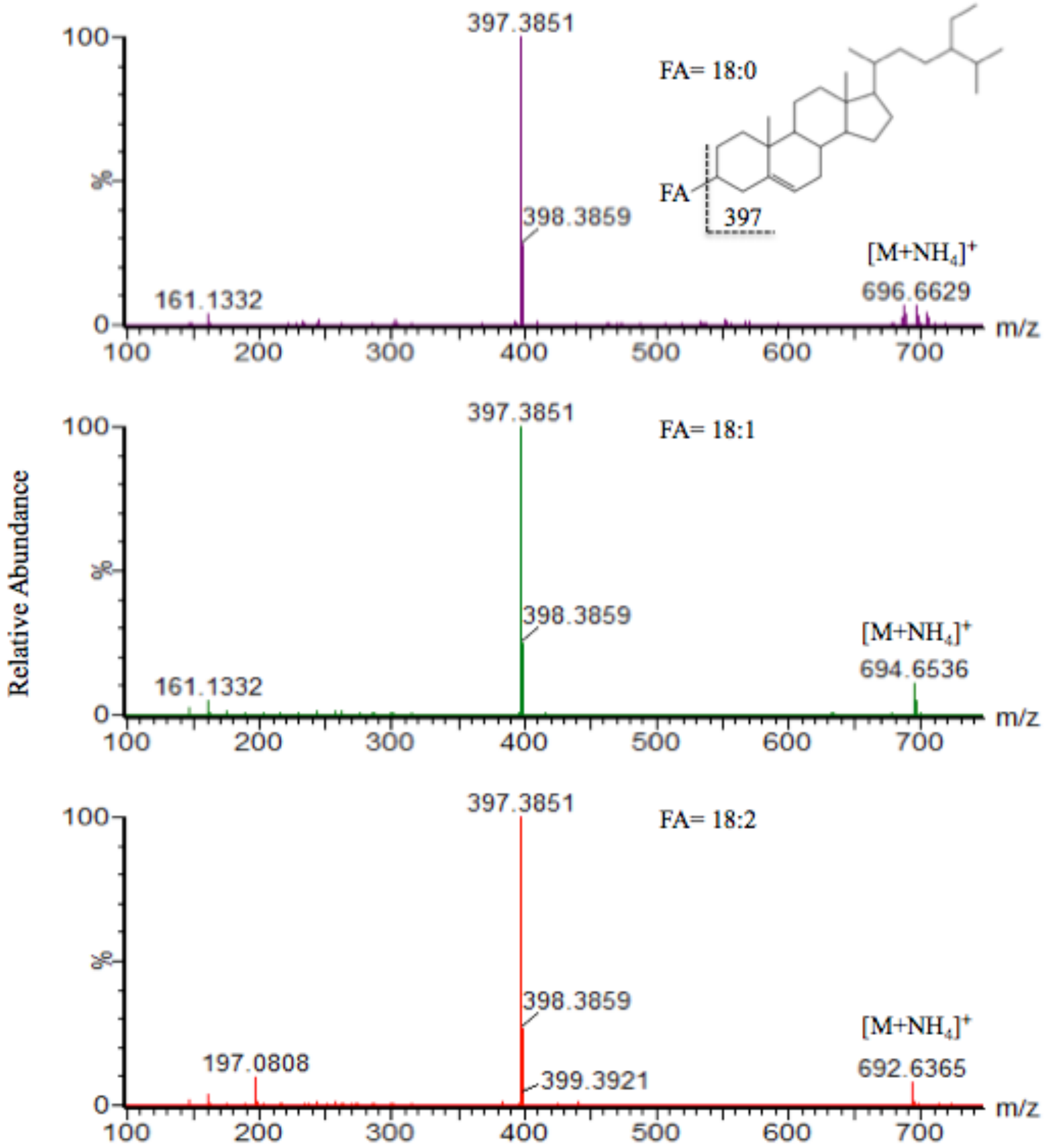

Figure S12. MS spectra of sitosterol esters: 18:0-sitosterol (top spectra), (B) 18:1-sitosterol (mid spectra), and 18:2-sitosterol (bottom spectra) in bilberry extract.

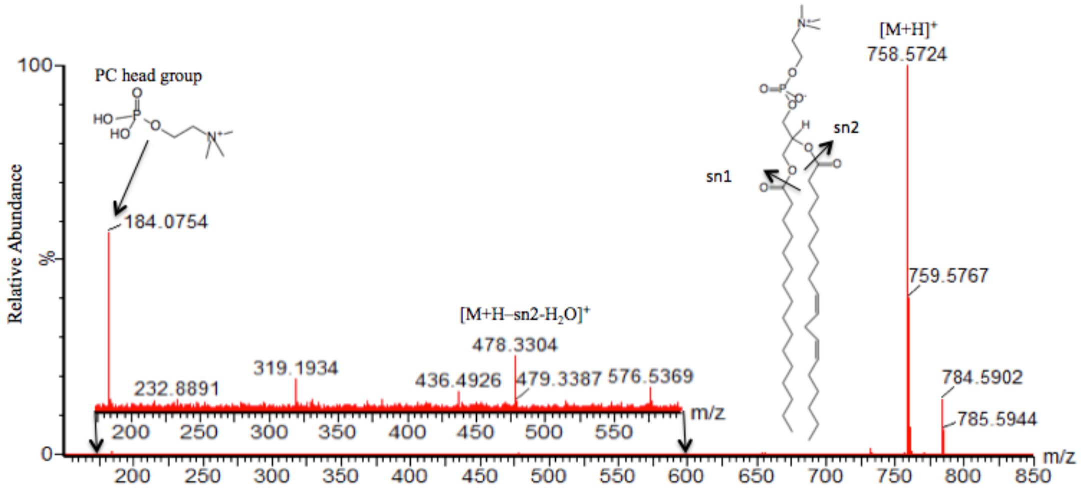

Figure S13. MS spectra of phosphatidylcholine (16:0/18:2) in bilberry extract.

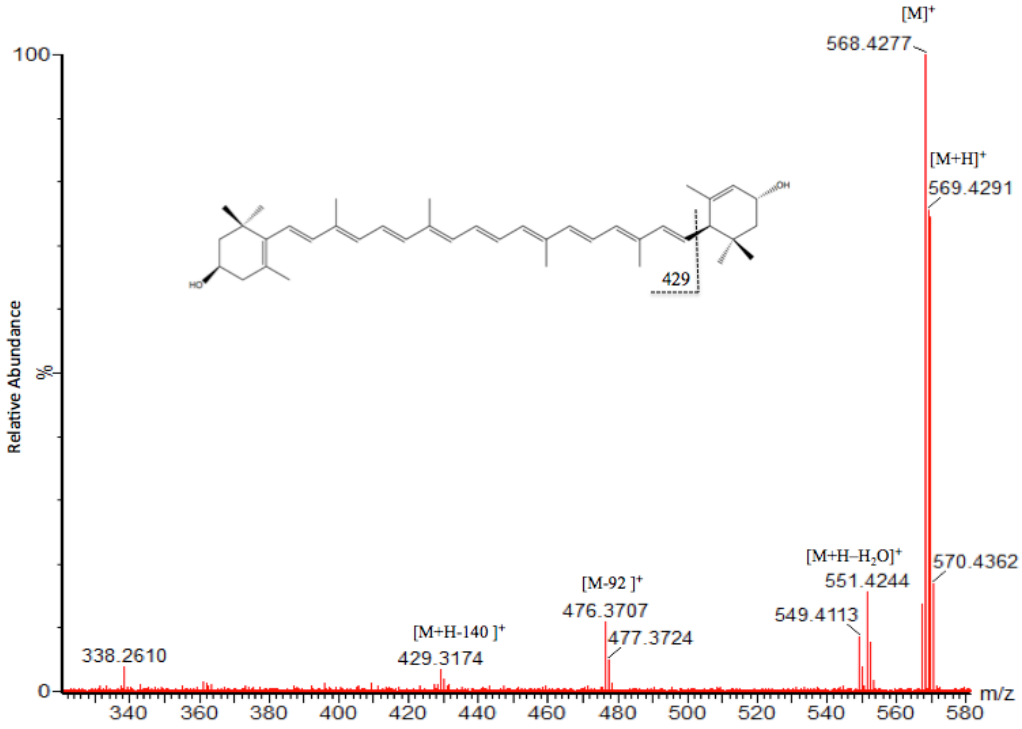

Figure S14. MS spectra of lutein in bilberry extract.
